# A qualitative investigation of Hispanic construction worker perspectives on factors impacting worksite safety and risk

**DOI:** 10.1186/1476-069X-10-84

**Published:** 2011-09-30

**Authors:** Cora Roelofs, Linda Sprague-Martinez, Maria Brunette, Lenore Azaroff

**Affiliations:** 1 Department of Work Environment, University of Massachusetts Lowell, Lowell, MA 01854, USA; 2 Community Health Program, Tufts University, Medford, MA 02155, USA

## Abstract

**Background:**

Hispanic workers have higher rates of injury and death on construction worksites than workers of other ethnicities. Language barriers and cultural differences have been hypothesized as reasons behind the disparate rates.

**Methods:**

We conducted two series of focus groups with union and non-union Hispanic construction workers to ask them about their perceptions of the causes for the unequal rates. Spanish transcripts were translated and coded in QSR NVivo software for common themes.

**Results:**

Workers reported a difficult work environment characterized by supervisor pressure, competition for jobs and intimidation with regard to raising safety concerns. Language barriers or cultural factors were not strongly represented as causative factors behind the rates.

**Conclusion:**

The results of this study have informed the development of an intervention trial that seeks to prevent falls and silica dust exposure by training contractors employing Hispanic construction workers in the elements of safety leadership, including building respect for their Hispanic workers and facilitating their participation in a safety program.

## Background

In the United States, Hispanic construction workers have had consistently higher work-related death rates compared to non-Hispanic construction workers [1]. In 2009, Hispanic workers experienced the highest rate of work-related fatal injuries at 3.7 incidents per 100,000 full time equivalent workers, compared to 3.4 for whites and 3.0 for blacks [2]. Dong's study [3] of more than 7,000 construction workers' medical records found that Hispanics were nearly 30% more likely to have medical conditions due to work-related injuries than white, non-Hispanics, after controlling for occupation, gender, age, and education. Their injuries were also more likely to be severe enough to cause lost workdays. Hispanic construction workers number almost 3 million and hold 30% of non-management jobs in construction [4]. These workers generally have limited-English language ability and lower education levels than other construction workers. They also are more likely to hold the more hazardous jobs within the construction industry. Despite a declining trend in work-related deaths overall, among Hispanic workers such fatalities continued to rise until the crash of the construction industry in 2009 [5].

Several investigators have examined, or have attempted to explain, the reasons for the disparity in injury rates. Language barriers, cultural differences, lack of safety training, economic disadvantage, lack of construction experience, and relegation to the most dangerous jobs within construction have all been suggested as reasons behind the rates [6-10]. Dong and Platner [1,3,11] note that Hispanics are over-represented in jobs with the more hazardous conditions laborers, helpers, and roofers face, and that "these dangerous occupations typically offer fewer opportunities for worker control of the work process, less on-the-job training, less job security, and lower average wages." One of the most prominent of the reports on Hispanic workers' greater risk cited "inadequate knowledge and control of recognized safety hazards and inadequate training and supervision of workers, often exacerbated by different languages and literacy levels of workers" as contributory factors based on federal and state investigations of the deaths of 200 Hispanic workers [7].

Language barriers, i.e., communication challenges resulting from lack of a common language between employers and employees, are often cited among the reasons for the higher rates and/or as a key factor to address in order to ameliorate the disparity. Menendez and Havea [8], in response to the growth in the proportion of construction fatalities among Hispanic construction workers, noted that Hispanic construction workers may need more language and literacy-appropriate safety training and information about their rights. Pransky et al. [9] hypothesized that the increased risk was potentially attributable to several factors, including: limited economic and political resources, language and cultural barriers, relegation to the most hazardous jobs, language difficulties, and workplace discrimination that may result in inadequate safety. However, in their study among non-agricultural Hispanic workers, they did not find that injured workers were less likely to speak English, to have received safety training, or to have received safety training in Spanish than non-injured Hispanic workers.

Goodrum and Dai [10] attributed higher Hispanic injury rates to the limited effectiveness of traditional training techniques due to Hispanic construction workers' lower education levels and more limited ability to understand English language and American culture. However, a language only becomes a barrier to safety training when training is provided. McGlothlin's survey [12] of Hispanic construction workers in New Orleans found that 57% had never received any safety training. O'Connor [13] assessed the adequacy of safety training provided to young Latino immigrant construction workers (14-21 years old) and found that, although the workers frequently performed tasks that posed significant injury risks, one quarter of respondents received no safety training at all, and approximately another quarter received less than an hour of training.

Forst et al's hospital-based interviews [14] with injured Hispanic workers (more than half of whom worked in construction) found that the injured workers provided the following explanations for their injuries: unsafe working conditions, including not understanding or being trained about the hazards, problems with personal protective equipment (not provided or defective), being overworked, and carelessness on their own part. Another hospital-based interview study found that Hispanic construction workers were more likely than blacks or whites to report that "nothing" could have prevented their injuries [15].

To better understand and address these issues, we conducted an exploratory qualitative research study as part of a larger community-based participatory research project. The project, Protección en Construcción: The Lawrence Latino Safety Partnership, is a National Institute for Occupational Safety and Health-funded study conducted by the University of Massachusetts Lowell, Laborers International Union of North America Local 175, the City of Lawrence, MA Mayor's Health Task Force and Lawrence Community Connections, a health and community development non-profit organization. Focus groups were conducted with both union and non-union workers to explore their perceptions of work-related hazards and potential factors associated with the disproportionate number of fatal and non-fatal accidents and exposures experienced by Latino construction workers (we use the terms "Latino" and "Hispanic" interchangeably). An additional aim of this research was to identify potential intervention elements. Finally, the intention of this work was to cultivate relationships with workers and to build community networks in preparation for the development of an intervention to reduce hazards. We have found focus groups to be successful in research aimed at building and strengthening community relationships (Sprague Martinez, L.S., Freeman, E., Peréa, F.C., From engagement to action: Assessing community readiness for disparities mobilization. Submitted manuscript).

## Methods

Two focus groups of three consecutive sessions for Hispanic construction workers - one for union and the other non-union workers - were held in April-May 2009. Participants came three nights in a row for two hours each night. This study used a modified convenience sample to select participants. A list of Spanish-speaking members of the labor union project partner was provided to University research staff who randomly selected workers from the list and invited 12 to participate. Eight workers participated in the union focus group at the Local's hall. Non-union Hispanic construction workers were recruited by the project's Hispanic community health outreach staff, the "promotores," who live and work in the Lawrence, MA community. The promotores identified potential participants through their community networks (e.g., neighborhoods, churches, family friends) and invited 12 to participate. Ten Hispanic construction workers participated in the non-union focus groups at a local senior center. All participants were male and were born in either Puerto Rico or the Dominican Republic. Focus group participants were given $75 for participating in the study over the three nights.

Participants authorized our recording of the sessions and agreed to participate through an oral consent process approved by the University of Massachusetts Lowell Institutional Review Board. All focus group discussions occurred in Spanish without English translation. One interviewer led the discussion from an interview guide that had been developed through our community-based participatory research process. That process included solicitation of desired themes from the project partners and other community members. The university researchers drafted the questions and translated them from English to Spanish, and these were again reviewed and revised by project members. The draft questions were piloted with three non-union construction members who considered the value and clarity of the questions. We again revised the interview guide, and it was approved by the project steering committee. In addition to the discussion leaders, a bilingual note taker participated in the focus groups, and served as the translator of the transcripts.

Our goal was to create an environment where questions could be answered honestly and deeply. Many comments and themes were repeated over the course of the three nights, despite the changing of the questions night to night. The first night's questions asked about falls they had experienced or knew about and causative factors. We probed further on the role of supervisors and contractors in health and safety on site, equipment and training. The second night's questions focused on prevention and safety practices and programs and how we could encourage participation in our intervention program. On the third night we asked about the role of others in making positive change, such as spouses, community organizations, and the United States Occupational Safety and Health Administration (OSHA).

Focus group recordings were transcribed in Spanish, and subsequently translated by the same person, who had also served as the note taker. No names were associated with comments, but the interviewer's comments were identified and letters were assigned to distinguish speakers. These translated conversations were input into QSR NVivo 9^® ^and coded based on the first author's impression of emergent themes, the themes of the interview guide, and the main research questions that concerned contributing factors to the cause of accidents and unsafe conditions on construction worksites. These theme codes were: why accidents happen, training, retaliation, speaking up, respect for workers, construction equipment, personal protective equipment, speed versus safety, supervisors, language issues, necessity for work, and responsibility for safety. Many quotes were multiply coded, reflecting the over-layering of the themes and experience of the participants. Quotes were selected that summarized other comments, were representations of a poignant sentiment, or described an uncommon view. The quotes shown below were edited from the English transcripts for grammar and spelling in English and written English.

## Results

### Why Accidents Happen/Speed versus Safety

Participants were presented with the fact of Hispanic construction workers' greater risk of falls and deaths on construction sites. They shared their perspective of the reasons for that disparate risk - primarily that Hispanic workers are under greater pressure to work fast, often to assure supervisors' bonuses for jobs completed early. They also felt that they have less recourse to protest and change dangerous conditions than non-Hispanic workers. They perceived "Americans," by which we assume they mean non-Hispanic United States-born workers, to have more knowledge of their rights, and more freedom to "work according to the hours," which we interpret to mean to work only the required hours at a pace dictated by the work. They did not universally blame contractors and supervisors for the increased risk. They took responsibility for "going along" or not taking precautions to protect themselves. Several sections presented below reflect the nuances of this key result.

Interviewer: The problem is that in Massachusetts falls are increasing among Hispanics and Brazilians.

Worker 1: You know why? Because of the pressure to work fast.

Worker 2: The harassment at work.

Worker 1: Doing the work faster, competing with others, and you don't care for yourself.

Worker 1: Americans have eight hours to complete a job, and what they do in eight hours Hispanic workers do in two and a half hours faster.

Worker 2: But when they're pressuring you to do the work...because if they don't pressure you, then you do the work right and at your pace.

Worker 1: If it's an eight hour job that's what you have. Eight hours to leave clean, complete and without injuries.

I know how to prevent falls. The best way to prevent falls, I'm going to explain to you [from] a case that I lived. So that there is no more falls is easy; but the point is how to get there. First of all, supervisors get bonuses, you understand? They get bonuses to finish the job faster. If the job is for two months, they'll finish it in one and a half. That is an offer for the supervisor. Naturally there will be pressure to finish the job, because the supervisor has that in his mind. Then he starts pressuring to work faster, and working faster means being less safe.

### Equipment and Personal Protective Equipment

Workers mentioned examples of where they found themselves (or co-workers) in dangerous situations because of not having the right ladder, or other appropriate equipment to work at heights.

Sometimes you have to do a job where the ladder doesn't reach and you have to put a piece of wood or a pole and go up there uncomfortable, and you could fall too.

They also described cases where they were given inadequate personal protective equipment (PPE) (such as dust masks instead of half-face respirators) or no equipment and using cloths to prevent breathing in dust. In contrast, some cited instances where supervisors were very concerned about PPE use, and even one case where a worker was fired for not using required PPE. Some workers described being required to provide their own PPE.

### Language and Literacy Issues

The inability to understand spoken English was not strongly articulated as a significant factor in construction safety by participants. Some workers raised the issue of inability to read signs - in Spanish -- as a potential problem.

*Interviewer*: *How do you communicate when you're learning to perform new tasks? Is it in Spanish or English?*

*Worker 1:No in Spanish.*

*Worker 2: There is always a foreman that speaks Spanish if you don't understand English that can explain how to do things.*

*Worker 3: Serves as interpreter.*

*Worker 1: Yes normally through the foreman, like he said, he explains and translates if necessary.*

*Interviewer:And all the jobs you have done are like that in the Boston area?*

*Worker 3:Normally yes.*

*Worker 2:Everywhere is the same, because the ones that don't know -- others help him until he gets use to it.*

I have noticed that a lot of people in this line of work don't even know how to read. That is a big problem, too. There are signs in Spanish, and they go in anyways because they can't read what the sign says, and that is a big problem and that causes lots of accidents.

### Training

Several comments reflected workers' cynicism about safety training, although at times, they also valued good training. Some felt that required training that they did receive was not helpful, either because it repeated concepts that they knew from working on the job, or was not relevant to actual safety risks, such as receiving first aid training when they were working with asbestos, or was difficult to apply on site, such as tying off when working on a ladder. They mentioned that they had known of cases where workers had bought the OSHA 10-hour safety training certificates that are required for work on public projects, but that there had been some greater scrutiny of that practice following the prosecution of a local fraudulent trainer. Tool box talks were mentioned, as well as forms that they had signed saying that they had been trained.

*Worker 1: Look, training is favorable, but no one is going to take 30 minutes to an hour to show you anything when all they want is to get the job done fast.*

*Worker 2:Yes, because they're not going to pay you to take a course on that. A lot of workers say "I don't need training. I just came to work to earn a living"*

I think that the OSHA has a great message. When I took the course they had a projector and they showed how everything was -- people climbing ladders that were the wrong size, people on scaffolds without harness. You know, they show you a lot of things.

### Responsibility for Safety

Participants spoke about how contractors' responsibility for a safe worksite is delegated to site personnel, and about their perspectives on workers' own responsibility for safety. Supervisors, union stewards, internal and consultant safety personnel, and the workers themselves, were identified as the people with responsibility for safety. Supervisors were identified as the site personnel with the greatest responsibility for the safety of the workers on the job. Workers spoke of being responsible for buying and maintaining their own safety equipment, of looking out for co-workers and hazards, and of their own and others' "negligence" as a cause of accidents. They also raised the issue of how the company's responsibility for safety is compromised by production pressures or undermined by contrary behavior by supervisors.

I think that everyone has to take care of themselves, my glasses, my helmet, and my gloves. Sometimes I've seen laborers talking to the foreman because he doesn't have safety equipment, then the example starts at home. How do you expect the workers to protect themselves?

The good thing is that between the workers you have to take care, you know? So you could be aware of not falling.

The supervisor should inspect the work before sending the worker. He is supposed to inspect and make sure everything is safe.

OSHA was at times described as having limited influence on a company's safety standards, and in other cases as having a significant influence on companies following an OSHA inspection and fine. Additionally, one worker saw that the contractor requirement (driven by state requirements, not federal OSHA as the worker believes) that workers have a 10-hour OSHA training card also supported workers in taking responsibility for safety.

OSHA requires every worker to have the card. If you don't have the card, then you're not accepted at work. OSHA designed that card so that you are conscious of what you're doing.

Temporary employment agencies were identified by workers as being both an important source of potential employment, and a source of potentially hazardous employment, and a means by which the contractor can avoid responsibility for safety.

I think that agencies are the skirt for the contractors. A contractor would rather call the agency to hire you, because that way they pay less and don't have the responsibility in case you get hurt.

### Supervisors

As seen from the sections above, focus group participants identified supervisors as playing a critical role in setting the expectations for safety on sites. In some cases, workers reported not even knowing who their employer was, only the supervisor. Workers spoke repeatedly of the negative impact on safety and the social environment resulting from supervisors' pressure on workers. They believed this pressure was motivated by opportunities for bonuses to supervisors who finished jobs under scheduled time. In some cases, participants spoke positively of the supervision that they had experienced and made note of some supervisors' attention to safety.

Worker 1: There is no defense for supervisors.

Worker 2: From a thousand, one that does things the way they should.

Worker 1: They don't care because they're interested in being the supervisor, and he works for the company, and the work is for two months, but he wants to do it in a month and a half so he can get a bonus and the worker -- who cares?[...] There are dangerous supervisors that think we are slaves. Sometimes they come and stand right behind you like a soldier when you're working.

Worker 2: They intimidate you.

Interviewer: If you could think of the ideal supervisor, what characteristics should this person have?

Worker 1: To be humane is the first thing. To be at the same level as the employee even though he has a higher rank. To acknowledge that the worker is a person just like him.

I worked once with a supervisor that was more conscientious. For example we would get together before starting the job and he would throw his speech. Before finishing he would make us line up and would take a good look at us, and who ever didn't have the correct boots he would say: you work today but if tomorrow you don't have the right boots, you better stay home.

Many workers said that they preferred working for "American" supervisors because what they perceive of as a perverse incentive for Hispanic supervisors to push workers in order to prove themselves.

That happens with the Hispanic supervisor that wants to be authoritarian -- to look good in front of the American that is coming behind him. So then what happens is that he over does it to look good. He pressures you. He wants to ensure a position there.

That's why I work with American supervisors. I'm convinced that American supervisors treat you better. They tell you, your safety is more important. You know? 'Take your time and do that the way it's supposed to be done.'

### Respect for Workers

The theme of the need for more respect for workers arose repeatedly among participants. This particular element of the social environment was deemed to be critical to improved safety and trust between supervisors and workers.

...that the workers can be seen as humans and not just as laborers [...] The boss, many times, is only looking at a person working, but doesn't give importance [to the worker] as a human being. Sometimes we're treated like dogs. Like animals. Then you feel resentment towards that person, and you don't want to share with them because they mistreat you. But if there is a little bit of consideration and trust then I will feel encouraged to share with them. But if you invite me, and treat me like an animal, I will never feel like even talking to you.

Worker 1: The problem is that the worker is so important and [yet] not important. He can do everything, but if employers don't like you, they never need you.

Worker 2: No one is indispensable at work.

Worker 3: Exactly. So no one is indispensable. You can be good or not, because you can leave and another one will come and do the job.

Worker 1: A supervisor where I'm working insulted me, and I had to confront him. I said not to treat me like a child, that under the suit and pants there was a man, and he said you're going to listen 'I'm your boss,' and I said, 'yes, you're my boss and I'm your employee.'

Worker 1: They treat you with wickedness.

Worker 2: Yes, the ones that receive bonuses if they finish the job earlier. When that happens [...] they abuse the employees.

Worker 3: They abuse us so much.

### Retaliation

Participants described an atmosphere of intimidation that prevented workers from speaking up about safety. They feared retaliation, most often in the form of getting fired or not offered future work, if they complained about a ladder or unsafe working conditions. They also pointed out that the ready availability of other workers to take their place created competition that resulted in workers taking safety risks such as doing hazardous work without training (e.g. assembling a scaffold). One worker described working with an injured hand for three days because a supervisor told him that he would lay him off and get another to replace him if he didn't want to work.

Worker 1: The worker is bad, too, because if he comes down, the other comes.

Worker 2: 'This one gets a ladder and goes up like nothing and this one is a softy.'

Worker 3: 'Look I have a guy, I'll bring him tomorrow.'

Worker 4: When you're leaving they say 'don't come tomorrow. We're reducing personnel.'

I was working at [a site in Boston] and a truck arrived with the scaffold assembly. Guys get it down then the supervisor asked another Hispanic supervisor: 'Listen, where is the scaffold? Take a couple of guys.' I haven't ever assembled that, but if they order me to do it, I have to do it, because otherwise they send me home.

If you express yourself they make you work with all the trash, and without protection, you understand?

Worker 1: Many times they're at work and no one says anything -- that's the problem -- that no one complains, and if no one complains, the problem will always be there.

Interviewer: And why you think no one complains?

Worker 1: Because they're afraid they may be fired.

Worker 2: You leave and another comes.

### Need for the Job

Participants repeatedly mentioned that Latino construction workers' need for the job - the necessity of paying work - as a causal factor in tolerating unsafe conditions. Only a few of the workers described having fulltime, year-round work. Many spoke of having only a few months of work a year. However, some workers saw also saw the need to refuse or resist the pull of "la necesidad."

At that moment, you're not thinking that you're risking your life. You are thinking that you have the necessity. Because of the necessity, you don't think that you can break a leg, but that you have to continue working.

Worker 1: The problem is that you know they're paying you, and because of the necessity you follow orders.

Worker 2: Yes, but sometimes the laborer has to speak up. Because sometimes they make you work in conditions that are not acceptable.

Worker 1: That depends on the person because if you have the necessity, you don't care.

Worker 3: Yes, because if you complain, they tell you right away don't come tomorrow, we'll call you and then, you have no job.

Worker 2: I understand but you have to have opposition.

Following an injury, one worker described his position:

I said to the guy, 'listen, I hurt my hand.' 'I'm sorry,' - this is the foreman, this is the supervisor, 'this job needs to be finished. If you don't want to finish, don't come tomorrow, and then we will call the union and have them send us another guy.' I didn't go to my house because I have three kids and my wife, and when you find work you have to take advantage of it. This doesn't happen all the time; I suffer three days with a white man, taking orders. My hand was bad. I went to the hospital. They told me I needed a specialist.

### Speaking Up

As described above, many workers felt that their only option in opposing unsafe conditions was to leave a job. A few workers' comments described a positive safety culture at some companies, however more often the "rules" discouraged participation by workers to improve conditions. There was discussion of the union, OSHA and rights protecting workers, but some workers felt that the point of opposition should be at the worksite, and that workers should not tolerate unsafe conditions.

Worker 1: If the person goes to the steward and reports what happened, and a report is done with the problems, and sent to the federal office where they're supposed to go, maybe this wouldn't happen. But no one reports it. Everyone complains, but does nothing. Because of the fear of losing the job.

Interviewer: And that is more common with Hispanic construction workers than non-Hispanics?

Worker 1: More with Hispanics. For Americans, that doesn't exist, because they know their rights. The majority of Hispanics don't know their rights. And even if they know their rights, they still don't complain, I'm telling you.

I would like to be a spokesman and help because I know there is a lot of injustice, even if I lose my job. I'm not living a bad life, nor am I living a rich life, but I can live without this job. Obvious I need it too. Work is work and you need the money, but I would like to be a spokesman if needed.

## Discussion

The analysis of the focus groups series that we held with Hispanic construction workers points to a tough safety climate on many of their worksites. Supervisor pressure to work fast and workers' necessity for work and fear of retaliation if they speak up about unsafe conditions were discussed by participants as the primary reasons behind Hispanic construction workers' disparate rates of injury on the job. Lack of training and language and cultural differences may play a role in contributing to these conditions, but were not prominently identified by the workers we interviewed. Although workers with limited English language skills may not be inclined to view their own limitation as a reason for accidents, workers did identify their responsibility for hazardous conditions, in particular by not "complaining." We interpret the findings above to suggest an analytical construct that Hispanic construction workers are more likely to face hazardous conditions that might lead to injury, because Hispanic construction workers, more than other workers, 1) face greater production pressures in a safety-challenged construction work environment, 2) are subject to disrespectful attitudes and intimidation, and 3) are more likely to tolerate unsafe conditions.

There are several potential limitations of this study. These data represent the views of a few individuals at a specific time and place. Limited qualitative data are not, as a rule, generalizable to larger populations. It is especially important to note that Hispanics cannot be characterized as a singular or uniform cultural, linguistic, economic or other social group. Additional limitations of the study include: lack of comparison of the data with alternative perspectives, possible errors of translation from Spanish to English, lack of verbal cues for interpretation, possible lingual and cultural misinterpretation, and unreflected bias of the interpreter of the results. We do believe, however, that these data and analysis are representative of the experiences of some Hispanic construction workers in the Northeast United States circa 2009.

There has been little exploration of the impact of a negative psychosocial environment on the rate of injuries in construction, although a recent study of workers in a variety of industries found association between job threat, harassment, and pressure, and higher rate of occupational injuries [16]. Zohar and others [17] have attributed lower occupational injury rates to better "safety climates" which include such constructs as demonstrated management commitment to safety and opportunities for meaningful employee participation in safety programs. Gillen et al. [18] found numerous challenges in evaluating relationships between safety climate and other psychosocial factors and injury severity in their study of injured construction workers. Indeed, the dynamic and overlapping potential factors affecting safety and organizational climate, hazardous conditions and workers' safety-related knowledge and actions, would prove challenging to evaluate in the construction sector.

Qualitative investigations of social phenomena are useful for unearthing the "why" behind what quantitative investigations have revealed to be the "what" - in this case, injury disparities in construction. Focus group findings' generalizability is limited, because the results are based on data from few non-randomly selected participants. Nevertheless, our results resonate with the findings of other investigators. Menzel et al. [19] conducted focus groups among union and non-union Latino construction workers in Nevada to elucidate their perceptions of the sources of risk in their jobs. They found many similar themes to those that arose in our study. The salient concepts of the origins of risk that they discuss include language barriers, lack of trade skills, and "traditional Latino values." The workers in their focus groups also identified economic pressure to work quickly due piecework pay or production deadlines, lack of appropriate tools and safety equipment, lack of or inadequate work tool and safety training, economic competition with other workers for scarce jobs, lack of sufficient skills in the chosen construction trade, exploitation, and immigration status as factors that contributed to risk and injury. Robertson's focus group study [20] of Latino construction workers exposed to noise identified the influence of ''traditional Latino values of collectivism, family focus, proscribed [sic] gender roles, and respect for authority figures'' on these workers' hearing protection behavior. Robertson's study also found that "participants agreed that Latino workers needed jobs and were willing to work in unsafe situations."

In some cases, these and other authors suggest that "traditional Latino values" such as respect for authority, hard work and prioritization of family interests over self-interest, play a role in either explaining Hispanics' greater risk, or can be taken advantage of to promote self-protective behaviors. However, given the context of the work environment described by the workers who participated in our focus groups, we question the explanatory role of "cultural" factors. Even if cultural factors play a role in reinforcing workers' response to pressure, removing the intimidation might also remove the influence of the traditional cultural value of "respect for authority." Palinkas and Arciniega [21] suggest that "traditional Latino values" held by an individual are unlikely to pose a barrier to economic advancement. They suggest that cultural norms change when confronted with new contexts in which the costs of maintaining those norms rises and the benefits of changing increase.

In introducing her paper "Hazardous Constructions of Latino Immigrants in the Construction Industry: The Case of a Post-Katrina New Orleans," Trujillo-Pagan [22] writes that many scholars and practitioners focus on "linguistically- and culturally-appropriate training and personal protective equipment and reproduce an emphasis on the worker rather than the employer." Her interviews with Latino construction workers in New Orleans found that "Latino workers believe they are racialized as 'hard workers' and that this racialization accounts for workplace discrimination. Specifically, Latino workers believed they were given more dangerous and risky work assignments because employers knew Latino workers would do the work... They assumed their ability to withstand a tough working situation was part of what they were paid for.." Additionally, the workers that she interviewed reported that "They had not received any training or information, and were unlikely to request this information from their employer because they did not believe their employer knew any more than they did. They were also concerned about their employer perceiving them as weak, unwilling to work, or as someone who *hace problemas*, i.e. makes problems; a "troublemaker."

How public health investigators and policy authorities interpret the causes of the disparate Latino risk for injury in construction will influence the design of interventions to address the problem. At the Latino Health and Safety Summit in April 2010 United States Secretary of Labor Linda Solis suggested that worker training was a central strategy to reduce Latino risk, and announced that OSHA would begin enforcing in earnest the OSHA policy that worker training must be provided in a way that is understandable to workers, i.e., in their language [23]. Programs that focus on worker training in Spanish, however, may not adequately address additional causative factors, such as the greater peril Hispanic workers may face in construction workplaces, and the challenges even well-trained and informed workers face in changing those conditions. (It is interesting to note that language barriers are hypothesized to be a barrier to safety, far more often than as a barrier to construction.) Interventions focused on changing hazardous conditions, rather than "hazardous traditional workers," would have the added benefit of addressing poor conditions for all workers, regardless of ethnicity or their personal values.

We are unaware of any interventions designed to shift contractor practices in order to lessen the risks faced by Hispanic workers on their worksites. However, some worker training programs have been designed specifically to address the needs of Hispanic workers [12,24]. In New Jersey, researchers developed and delivered a hazard awareness training program targeting Latino immigrant day laborers, many of whom were working in construction. Both qualitative and quantitative pre- and post-training assessments demonstrated increased knowledge and "self-protective actions" among trainees. The authors conclude that "participatory, peer-lead training...may have a positive effect...but major changes would require employer engagement" [25].

## Conclusion

These focus group findings suggest that Hispanic construction workers in the United States perceive a tough safety climate and hazardous conditions. Rather than focus on the workers as the target of change, we designed an intervention for contractors employing Hispanic construction workers. The intervention program, Leaders in Safe Construction (LISC), includes multiple steps and coaching for contractors to enhance their ability to assure safety for all workers. LISC includes a supervisor training component aimed at improving their management skills, and increasing their understanding and respect for their workers. More details on this program can be found at http://www.lawrenceworksafe.org. We believe that widespread and effective alteration in the high injury rates experienced by immigrant Hispanics working in construction in the United States, may be affected by enhanced safety climates and reduced hazards. While Spanish-language training for workers is necessary, it may not translate into improved conditions. Indeed, the key may be better enforcement of workplace standards and targeted training of those with the power over these conditions: construction employers.

## List of Abbreviations

LISC: Leaders in Safe Construction; OSHA: U.S. Occupational Safety and Health Administration; PPE: personal protective equipment.

## Competing interests

The authors declare that they have no competing interests.

## Authors' contributions

CR helped design the study, conducted the analysis and drafted the manuscript. LSM contributed to study design and analysis strategy and drafted the parts of the manuscript related to methodology. LA helped design the study, directed the overall research project, and contributed to the interpretation of the results and the writing of the manuscript. MB helped design the study and contributed to the interpretation of the results and the writing of the manuscript. All authors read and approved the final manuscript.
